# Proteomic characterisation of bovine and avian purified protein derivatives and identification of specific antigens for serodiagnosis of bovine tuberculosis

**DOI:** 10.1186/s12014-017-9171-z

**Published:** 2017-11-02

**Authors:** José Antonio Infantes-Lorenzo, Inmaculada Moreno, María de los Ángeles Risalde, Álvaro Roy, Margarita Villar, Beatriz Romero, Nieves Ibarrola, José de la Fuente, Eugenia Puentes, Lucía de Juan, Christian Gortázar, Javier Bezos, Lucas Domínguez, Mercedes Domínguez

**Affiliations:** 1 Centro Nacional de Microbiología, Instituto de Salud Carlos III, Unidad de Inmunología Microbiana, Majadahonda, Madrid Spain; 2 VISAVET Health Surveillance Centre, Universidad Complutense de Madrid, Madrid, Spain; 3 SaBio Instituto de Investigación en Recursos Cinegéticos IREC (CSIC-UCLM-JCCM), Ciudad Real, Spain; 4 MAEVA SERVET S.L, Alameda del Valle, Madrid Spain; 5 Departamento de Sanidad Animal, Facultad de Veterinaria, Universidad Complutense de Madrid, Madrid, Spain; 6 Unidad de Proteómica, Instituto de Biología Molecular y Celular del Cáncer—USAL-CSIC, ProteoRed ISCIII, Campus Unamuno, Salamanca, Spain; 7 CZ Veterinaria S.A, Porriño, Pontevedra Spain; 8 Department of Veterinary Pathobiology, Center for Veterinary Health Sciences, Oklahoma State University, Stillwater, OK USA

**Keywords:** Tuberculosis, PPD, P22, Proteome, Cross-reaction

## Abstract

**Background:**

Bovine purified protein derivative (bPPD) and avian purified protein derivative (aPPD) are widely used for bovine tuberculosis diagnosis. However, little is known about their qualitative and quantitative characteristics, which makes their standardisation difficult. In addition, bPPD can give false-positive tuberculosis results because of sequence homology between *Mycobacterium bovis* (*M. bovis*) and *M. avium* proteins. Thus, the objective of this study was to carry out a proteomic characterisation of bPPD, aPPD and an immunopurified subcomplex from bPPD called P22 in order to identify proteins contributing to cross-reactivity among these three products in tuberculosis diagnosis.

**Methods:**

Trypsin digests of bPPD, aPPD and P22 were analysed by nanoscale liquid chromatography-electrospray ionization tandem mass spectrometry. Mice were immunised with bPPD or aPPD, and their serum was tested by indirect ELISA for reactivity against these preparations as well as against P22.

**Results:**

A total of 456 proteins were identified in bPPD, 1019 in aPPD and 118 in P22; 146 of these proteins were shared by bPPD and aPPD, and 43 were present in all three preparations. Candidate proteins that may cause cross-reactivity between bPPD and aPPD were identified based on protein abundance and antigenic propensity. Serum reactivity experiments indicated that P22 may provide greater specificity than bPPD with similar sensitivity for ELISA-type detection of antibodies against *M. tuberculosis* complex.

**Conclusion:**

The subpreparation from bPPD called P22 may be an alternative to bPPD for serodiagnosis of bovine tuberculosis, since it shares fewer proteins with aPPD than bPPD does, reducing risk of cross-reactivity with anti-*M. avium* antibodies.

**Electronic supplementary material:**

The online version of this article (10.1186/s12014-017-9171-z) contains supplementary material, which is available to authorized users.

## Background

Bovine tuberculosis, caused mainly by *Mycobacterium bovis* (*M. bovis*), is a serious zoonotic infectious disease in cattle that can be also transmitted to humans [1]. Tuberculosis in cattle is subjected to compulsory eradication programmes based on the test-and-slaughter policy and results in a significant economic impact derived from a decreased production, trade restrictions and increased mortality rates in the infected herds [2, 3].

In Spain, the single intradermal tuberculin (SIT) test and interferon-gamma (IFN-γ) assay are used to diagnose bovine tuberculosis in official eradication programmes. Both tests depend on cell-mediated immune responses triggered by Th1 lymphocytes and a bovine purified protein derivative (bPPD) obtained from *M. bovis*. While bPPD is useful, it contains antigens shared by non-tuberculous mycobacteria and mycobacterial disease vaccines [4–7], giving rise to immune cross-reactions that limit its diagnostic specificity. For this reason, vaccination of cattle against mycobacterial diseases such as tuberculosis and paratuberculosis is prohibited in countries running tuberculosis eradication programmes. For the same reason, the diagnostic reliability of recommended tuberculosis tests can be improved by comparing reactivity against bPPD with reactivity against avian purified protein derivative (aPPD), derived from *M. avium*. However, this comparative testing requires additional time, reagents and labor. In addition, it can reduce overall sensitivity, since animals that show greater immunoreactivity against aPPD than bPPD are often judged negative for tuberculosis when in fact they are infected with *M. bovis* [8, 9].

While standard tuberculosis tests focus on cell-mediated immune responses, recent work highlights the importance of humoral responses. Early immune responses in bovine tuberculosis are dominated by cell-mediated immunity. However, some infected animals may have an antibody response in the absence of cell-mediated responses, particularly when the bacterial load is high [10, 11]. Therefore, researchers have been developing serological assays as diagnostic tests to detect infected animals missed by skin tests and the IFN-γ assay [12–15]. Serological tests are simple and inexpensive and can be used to screen many animals in a short time. Preparations of bPPD and P22 have been used as coating antigens in serological immunoassays to detect infected domestic and wild animals [16–20].

Improving diagnostic tests based on bPPD and aPPD requires detailed understanding of their protein composition, which would allow these reagents to be standardised and further optimised to reduce cross-reactivity. However, the composition of both reagents is poorly understood, and available data are to some extent contradictory [21, 22]. Proteomic analysis of bPPD and aPPD used in the UK and Brazil [22] identified 116 proteins in two bPPD preparations and 87 in two aPPD preparations; 32 proteins were shared between bPPDs and aPPDs. A similar study of a bPPD preparation used in South Korea [21] identified 271 proteins; 33 were also present in the previously analysed preparations from the UK and Brazil, and 15 were T cell antigens that induce cell-mediated immune responses detectable by standard diagnostic tests. These results provide molecular insights into possible false positives due to bPPD, since the T cell antigens showed an average sequence similarity of 78% with *M. avium* and 74% with *M. paratuberculosis* proteins.

Given the usefulness of proteomic analysis of bPPD and aPPD, we studied preparations from CZ Veterinaria widely used in European bovine tuberculosis eradication programmes. We also performed proteomic analysis of P22, a novel protein complex affinity-purified from the bPPD preparation for the first time, which may serve as an alternative antigen in tuberculosis immunodiagnosis.

## Methods

### Ethics statement

All animal experiments in this study were conducted according to Spanish regulations (RD 53/2013) and European regulations (EU Directive 2010/63/EU). All animal procedures were approved by the Ethics Committee of the Instituto de Salud Carlos III (permit CBA22_2014-v2) and by the Community of Madrid (permit PROEX 278/14).

### Immunopurification of P22

BALB/c mice were hyperimmunised with bPPD (CZ Veterinaria, Porriño, Spain), giving rise to a hybridome that secretes a specific monoclonal antibody against an epitope shared by two proteins, MPB70 and MPB83, which form part of a multiprotein complex within bPPD. This monoclonal antibody was conjugated to a HiTrap NHS-activated HP column (GE Healthcare, Little Chalfont, UK) according to the manufacturer’s protocol. The column was loaded with bPPD and the complex containing MPB70 and MPB83, which we named P22, was immunopurified (this process has been patented under patent EP16382579, “Methods and compositions for tuberculosis diagnosis”).

### Sample preparation for proteomic analysis

Batches of bPPD prepared from *M. bovis* strain AN5 and of aPPD prepared from *M. avium* strain D4 ER were obtained from CZ Veterinaria. P22 was obtained as described above. Each sample (bPPD, aPPD and P22) was analysed in three biological replicates. The protein mixtures were precipitated using trichloroacetic acid/acetone, and total protein concentration was determined using a Pierce protein assay at 660 nm (ThermoFisher Scientific, MA, USA). Protein pellets (20 µg) were resuspended and denatured in 20 µl of 7 M urea/2 M thiourea/100 mM TEAB (pH 7.5), then reduced with 2 µl of 50 mM Tris (2-carboxyethyl) phosphine (pH 8.0, AB SCIEX) at 37 °C for 60 min, and finally incubated for 10 min at room temperature with 2 µl of 200 mM methyl methanethiosulfonate (ThermoFisher Scientific) to block cysteines. To reduce urea concentration, samples were diluted to a final volume of 70 µl using 25 mM TEAB.

Proteins were digested overnight at 37 °C with sequencing-grade modified trypsin (Sigma-Aldrich, MO, USA) added in a trypsin:protein (w/w) ratio of 1:20. Digestion was stopped by adding 1% trifluoroacetic acid, and reactions were desalted by passage through StageTip C18 Pipette tips (ThermoFisher Scientific) [23]. Desalted eluates were dried-down and stored until proteomic analysis.

### Liquid chromatography and mass spectrometry

Protein digests (1 µg) were analysed by one-dimensional nanoscale liquid chromatography-electrospray ionization tandem mass spectrometry on an Eksigent nanoLC Ultra 1D plus (AB SCIEX) coupled to a 5600 Triple TOF^®^ mass spectrometer (AB SCIEX) equipped with a Nanospray III source. The analytical column was a silica-based reverse-phase Waters Acquity UPLC^®^ M-Class Peptide BEH C18 column (75 µm × 150 mm, 1.7 µm particles, 130 Å pore). The trap column was an Acclaim C18 PepMap™ 100 (100 µm × 2 cm, 5 µm particles, 100 Å pore; ThermoFisher Scientific), which was connected ahead of the analytical column. The loading pump delivered a solution of 0.1% formic acid in water at 2 µl/min. The nano-pump provided a flow-rate of 250 nl/min and was operated under gradient elution conditions. Peptides were separated using a gradient ranging from 2 to 90% mobile phase B (mobile phase A: 2% acetonitrile, 0.1% formic acid; mobile phase B: 100% acetonitrile, 0.1% formic acid). The gradient lasted 250 min in the case of bPPD and aPPD, and 30 min in the case of P22. The injection volume was 5 µl.

Data were acquired on the TripleTOF system using the following operating parameters: ion-spray voltage floating, 2300 V; curtain gas, 35; interface heater temperature, 150; ion source gas 1, 25; and declustering potential, 100 V. Data were acquired in data-dependent acquisition (DDA) mode using Analyst TF 1.7 software (AB SCIEX). A mass spectrometry survey scan (0.25 s) over the mass range 350–1250 Da was followed by 35 tandem mass spectrometry scans (100 ms) over the mass range 100–1800 Da, giving a total cycle time of 4 s. Switching criteria were defined as the presence of ions at an abundance of > 90 counts per sec with charges of 2–5 and 350 < *m/z* < 1250. Former target ions were excluded for 15 s. Collision energy was controlled using a DDA rolling collision energy script.

### Proteomic data analysis

Data from mass spectrometry and tandem mass spectrometry were processed using Analyst^®^ TF 1.7 software (AB SCIEX). Raw data were converted into mgf files, which were searched against the NCBI RefSeq protein databases for *M. bovis* AN5 in the case of bPPD and P22 (15,834 entries in May 2016) or for *M. avium* subsp*. avium* ATCC 25291 in the case of aPPD (8712 entries in May 2016). These searches were conducted using Mascot Server 2.5.1 (Matrix Science, London, UK), and they included the corresponding reversed entries. Searches were conducted with methylthiolation (C) as a fixed modification and with the following variable modifications: acetyl (Protein N-term), Gln to pyro-Glu (N-term Q), Glu to pyro-Glu (N-term E) and Oxidation (M). Peptide mass tolerance was 25 ppm, fragment mass tolerance was 0.05 Da, and 2 missed cleavages were allowed. The false discovery rate (FDR) had to be ≤ 1% for peptide identification to be considered successful.

The method of spectral counts [24] was used to assess relative abundance of different proteins in bPPD, aPPD and P22. Spectral counts for different proteins were first normalised by dividing them by the total spectral counts of the sample [25]. Relative abundance was represented as the mean of the replicates ± standard deviation (SD). Average antigenic propensity (AAP) of proteins was determined using the Kolaskar and Tongaonkar method [26] (http://imed.med.ucm.es/Tools/antigenic.pl) in order to identify proteins likely to elicit an antibody response. If the entire protein showed AAP > 1.0, then all amino acid residues within the protein with AAP > 1.0 were considered likely to be antigenic. Sequences of proteins were compared against those in the NCBI database using BLAST searches (http://blast.ncbi.nlm.nih.gov/Blast.cgi). Venn diagrams were prepared using an open-access plotter (http://omics.pnl.gov/software/VennDiagramPlotter.php).

### Indirect ELISA

Four female BALB/c mice were immunised with three inoculations of bPPD and another four mice with three doses of aPPD. These inoculations were administered at least 2 weeks apart. Sera from the mice were assayed for reactivity against bPPD, aPPD or P22 using an in-house indirect ELISA as follows. Plates were coated with bPPD, aPPD or P22 at 10 μg/ml, then blocked with 2% bovine serum albumin in phosphate-buffered saline (PBS) and washed with PBS containing 0.05% Tween 20 (PBST). Serial twofold dilutions of serum (starting at 1:8) were prepared and assayed in triplicate, with ELISA plates incubated for 1 h at 37 °C. Next, horseradish peroxidase-conjugated goat anti-mouse IgG antibody (SouthernBiotech, AL, USA) was added, and colour was developed by adding o-phenylenediamine substrate (OPD, Sigma–Aldrich). The reaction was stopped using 3 N H_2_SO_4_. Optical density was measured at 492 nm. Negative control serum was pooled from four female BALB/c mice that were not immunised.

## Results and discussion

Bovine tuberculosis is a serious problem for public health and animal health, and the relative lack of specificity and sensitivity of current standard diagnostic tests based on bPPD and aPPD means that some animals showing false positive results for tuberculosis infection are needlessly slaughtered, while animals showing false negative results are spared and may pose a threat to disease control and eradication. Improving bPPD as a diagnostic agent has become even more important as serological tuberculosis tests become more attractive than SIT and IFN-γ assays.

Here we provide detailed proteomic descriptions of bPPD and aPPD and identify components common to both preparations. These results may help guide efforts to improve the diagnostic performance of bPPD and, in particular, reduce cross-reactivity. Finally, we propose a less complex bPPD-derived immunoproduct more amenable to standardisation and less likely to cross-react with antibodies against *M. avium* proteins, which may serve as a substitute for bPPD in immunodiagnostic tests against tuberculosis.

### Proteins in bPPD, aPPD and P22

We identified 2678 peptides in bPPD, 6465 in aPPD and 492 in P22. We then reconstructed the proteins present in each preparation and we included only proteins that (1) were represented by at least two peptides in each replicate where they were present, and (2) were present in at least two of the three replicates. In this way, our study identified 456 proteins in bPPD (Additional file 1), 1019 in aPPD (Additional file 2) and 118 in P22 (Additional file 3). The proteins identified in bPPD correspond to at least 8.5% of the coding sequences in the *M. bovis* genome; the proteins in aPPD, to at least 23.3% of the *M. avium* subsp. *avium* coding sequences; and the proteins in P22, to 1.5% of *M. bovis* coding sequences.

We were able to identify far greater numbers of proteins in bPPD and aPPD than previous studies, even though we used the same source strains for bPPD and aPPD as those studies. Studies of bPPD in UK, Brazil and South Korea identified only 104, 49 and 271 proteins, respectively [21, 22]. Studies of aPPD in UK and Brazil found only 63 and 57 proteins, respectively [22]. These discrepancies may reflect differences in how the PPDs were obtained from bacterial culture, as well as hardware and procedural differences in how proteomics data were obtained (e.g. mass spectrometer model, liquid chromatography flow rate, in-gel versus in-solution digestion), and how proteomics data were searched against the databases.

Tables 1, 2 and 3 show the 10 most abundant proteins in bPPD, aPPD and P22, respectively. These account for approximately 50% of total proteins determined in bPPD and P22 but no more than 15% in aPPD. These results imply that the vast majority of antibodies induced by *M. bovis* infection should be detectable in a serodiagnostic test based on bPPD or P22. Our estimates of relative protein abundances in the three preparations were obtained without labelling, which means that they are influenced by the proteomics methodology, particularly the duration of the dynamic exclusion process [25, 27]. Label-free abundances will also underestimate actual abundance in mixtures in which one or a few proteins dominate strongly. Thus, our abundance estimates are likely to be relatively accurate in the case of aPPD, in which no protein accounts for more than 2.7% of total protein, but they are likely to be underestimated in the case of bPPD, in which the ESAT-6-like protein EsxB (CFP-10) accounts for 12.2% of total protein, and in the case of P22, in which MPB70 accounts for 26.0% of total protein. The high abundance of MPB70 in *M. bovis* and its immunological properties suggest that replacing bPPD with P22 may increase the sensitivity of bovine tuberculosis immunological diagnosis [28].

**Table 1:** Ten most abundant proteins in bPPD identified by liquid chromatography-mass spectrometry

No.	NCBI accession	Name	Relative abundance (%) Mean ± SD	AAP
1	489495023	ESAT-6-like protein EsxB	12.2 ± 1.1	0.9934
2	489495046	6 kDa early secretory antigen target	11.4 ± 2.0	0.9935
3	489509783	Cell surface protein MPB70	8.5 ± 0.9	1.0478
4	489513185	Molecular chaperone GroES	3.7 ± 0.1	1.0245
5	489509769	Cell surface protein MPB83	3.4 ± 0.1	1.0336
6	489497323	Molecular chaperone GroEL	3.0 ± 0.2	1.025
7	489516779	ESAT-6-like protein EsxL	2.21 ± 0.1	1.0118
8	489503953	ESAT-6-like protein EsxN	2.1 ± 0.0	1.0193
9	489504801	Hypothetical protein MPB63	2.0 ± 0.0	1.0332
10	489505308	Alpha-crystallin	1.6 ± 0.3	1.0135

*AAP* average antigenic propensity

**Table 2:** Ten most abundant proteins in aPPD identified by liquid chromatography-mass spectrometry

No.	NCBI accession	Name	Relative abundance (%) Mean ± SD	AAP
1	489973362	Bacterioferritin	2.7 ± 1.6	1.0226
2	497662805	ModD protein, partial	1.9 ± 0.4	1.0170
3	497665169	Molecular chaperone GroEL	1.6 ± 0.2	1.0244
4	500044420	PPE family protein	1.5 ± 0.3	1.0271
5	489970321	50S ribosomal protein L7/L12	1.4 ± 0.0	1.0327
6	564987547	Acyl dehydratase	1.4 ± 0.7	1.0457
7	489970303	Elongation factor Tu	1.3 ± 0.4	1.0226
8	489970531	Hypothetical protein	1.2 ± 0.2	1.0136
9	4566238	10 kDa heat shock protein	1.1 ± 0.1	1.0261
10	489973377	Hypothetical protein	1.1 ± 0.1	1.0157

*AAP* average antigenic propensity

**Table 3:** Ten most abundant proteins in P22 identified by liquid chromatography-mass spectrometry

No.	NCBI accession	Name	Relative abundance (%) Mean ± SD	AAP
1	489509783	Cell surface protein MPB70	26.0 ± 2.6	1.0478
2	489509769	Cell surface protein MPB83	5.4 ± 0.8	1.0336
3	489495046	6 kDa early secretory antigen target	4.4 ± 2.3	0.9935
4	489505073	Hypothetical protein	2.6 ± 0.1	1.0401
5	489497323	Molecular chaperone GroEL	2.4 ± 0.6	1.0250
6	489495023	ESAT-6-like protein EsxB	2.3 ± 0.3	0.9934
7	489498552	Elongation factor Tu	2.0 ± 0.2	1.0225
8	489505308	Alpha-crystallin	1.9 ± 0.7	1.0135
9	489501012	5-Methyltetrahydropteroyltriglutamate–homocysteine methyltransferase	1.8 ± 0.5	1.0402
10	489504801	Hypothetical protein MPB63	1.4 ± 0.1	1.0332

*AAP* average antigenic propensity

### Proteins shared among bPPD, aPPD and P22

**Fig. 1:**
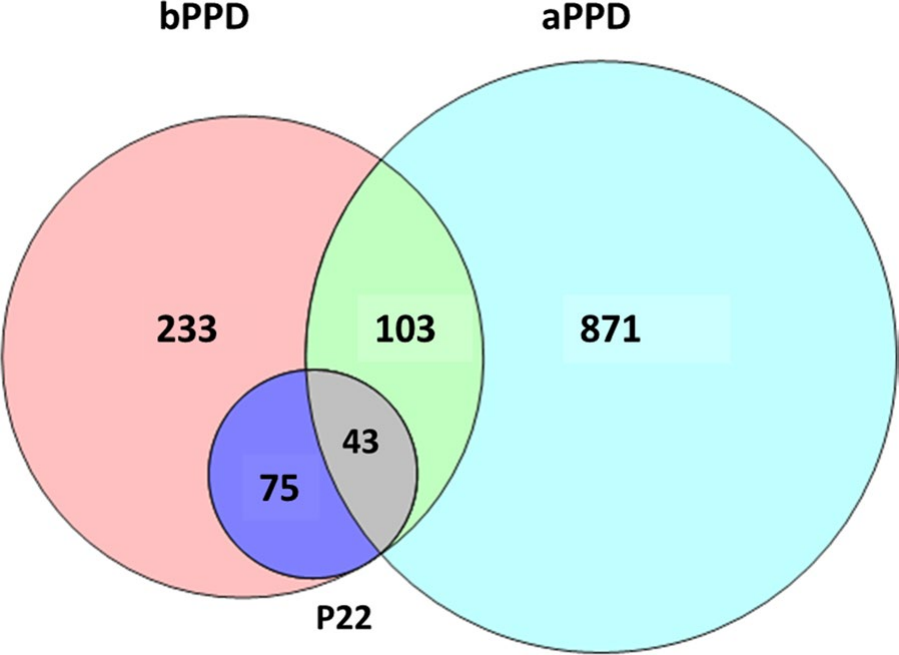
Identification of proteins shared among bPPD, aPPD and P22

A total of 146 proteins were found to be common to bPPD and aPPD, accounting for 32 and 14.3% of total proteins in the respective preparations. Of these 146 proteins, only 43 were present in P22 (Fig. 1). The numbers of these proteins with relative abundances > 0.1% based on spectral counting were 61 in bPPD, 92 in aPPD and 43 in P22 (Additional file 4). Estimation of the ability of these proteins to induce a B cell-mediated immune response [26] identified 32 that we predict contribute to cross-reactivity (Table 4), either because they show an abundance ≥ 0.5% in at least one of the two PPDs or because they have AAP ≥ 1.04. Only 21 of these 32 proteins are present in P22 (Table 4), suggesting that it may provide greater diagnostic specificity than bPPD.

**Table 4:** Putative proteins shared among bPPD, aPPD and P22 that may contribute to cross-reactivity

No.	NCBI accession	Description	Relative abundance in % Mean ± SD			AAP		Ident/query coverage
			bPPD	aPPD	P22	bPPD	aPPD	
1	489513185 *4566238*	Molecular chaperone GroES *10* *kDa heat shock protein*	3.6 ± 0.0	1.1 ± 0.1	1.0 ± 0.3	1.0243	1.0261	97/100
2	489497323 *497665169*	Molecular chaperone GroEL *Molecular chaperone GroEL*	3.0 ± 0.2	1.6 ± 0.2	2.5 ± 0.6	1.0251	1.0244	94/100
3	489516779 *489971186*	ESAT-6-like protein EsxL *Hypothetical protein*	2.2 ± 0.1	0.7 ± 0.2	1.4 ± 0.3	1.0124	1.0238	87/100
4	489503953 *489971186*	ESAT-6-like protein EsxN *Hypothetical protein*	2.1 ± 0.0	0.7 ± 0.2	1.2 ± 0.4	1.0193	1.0238	94/100
5	489496900 *497665296*	Molecular chaperone DnaK *Molecular chaperone DnaK*	1.5 ± 0.1	0.8 ± 0.2	0.9 ± 0.1	1.0157	1.0149	95/94
6	489500428 *489971186*	ESAT-6-like protein EsxI *Hypothetical protein*	1.4 ± 0.1	0.7 ± 0.2	–	1.0154	1.0238	87/100
7	489498552 *489970303*	Elongation factor Tu *Elongation factor Tu*	1.2 ± 0.0	1.3 ± 0.4	2.0 ± 0.2	1.0225	1.0226	97/100
8	489506691 *489969124*	Meromycolate extension acyl carrier protein *Acyl carrier protein*	1.0 ± 0.1	0.7 ± 0.3	–	1.0236	1.0243	92/100
9	499253161 *48997118*6	Secretion protein *Hypothetical protein*	0.7 ± 0.2	0.7 ± 0.2	–	1.0113	1.0238	93/100
10	489506256 *489968974*	Cell wall synthesis protein Wag31 *DivIVA domain*-*containing protein*	0.7 ± 0.1	0.7 ± 0.2	1.0 ± 0.4	0.9988	1.0052	86/100
11	489501012 *497661234*	5-Methyltetrahydropteroyltriglutamate *5*-*Methyltetrahydropteroyltriglutamate*	0.6 ± 0.0	0.1 ± 0.0	1.8 ± 0.5	1.0388	1.0374	86/99
12	489500440 *489971185*	peptidase M22 peptidase M22	0.5 ± 0.0	0.7 ± 0.3	0.6 ± 0.2	0.9816	0.9844	87/100
13	489498442 *489970321*	50S ribosomal protein L7/L12 *50S ribosomal protein L7/L12*	0.5 ± 0.0	1.4 ± 0.0	–	1.0392	1.0327	93/100
14	489504514 *489973362*	Bacterioferritin *Bacterioferriti*n	0.5 ± 0.1	2.7 ± 1.6	0.8 ± 0.1	1.0193	1.0226	88/100
15	489495992 *497665638*	Diacylglycerol acyltransferase *Diacylglycerol acyltransferase*	0.5 ± 0.1	0.6 ± 0.0	0.9 ± 0.3	1.0142	1.0141	85/95
16	489504572 *500042691*	Diacylglycerol acyltransferase *Diacylglycerol acyltransferase*	0.5 ± 0.01	0.9 ± 0.1	1.0 ± 0.1	1.0166	1.0192	81/95
17	489998054 *656316315*	Serine protease *Peptidase S1*	0.5 ± 0.0	0.9 ± 0.2	–	1.0357	1.0264	73/96
18	489503193 *497663153*	Universal stress protein *Universal stress protein*	0.4 ± 0.0	0.6 ± 0.0	0.4 ± 0.0	1.0434	1.0431	88/100
19	489513178 *497664719*	Molecular chaperone GroEL *Molecular chaperone GroEL*	0.4 ± 0.0	0.7 ± 0.1	0.6 ± 0.1	1.0416	1.0399	84/98

No.	NCBI accession	Description	Relative abundance (%) ± SD			AAP		Ident/query coverage
				aPPD	P22	bPPD	aPPD	
20	554796584 *656316442*	aconitate hydratase *aconitate hydratase*	0.3 ± 1.0	0.6 ± 0.2	1.0 ± 0.2	1.0252	1.0245	89/99
21	489509166 *497663814*	Hypothetical protein *Hypothetical protein*	0.3 ± 0.0	0.7 ± 0.2	–	1.0146	1.0152	87/100
22	489997754 *500042691*	diacylglycerol acyltransferase *diacylglycerol acyltransferase*	0.2 ± 0.1	0.9 ± 0.1	1.0 ± 0.1	1.0225	1.0188	83/96
23	489501803 *497661528*	ATP synthase subunit beta *F0F1 ATP synthase subunit beta*	0.2 ± 0.0	0.6 ± 0.1	0.3 ± 0.3	1.0239	1.0238	95/98
24	489496308 *564987547*	Acyl dehydratase *Acyl dehydratase*	0.1 ± 0.1	1.4 ± 0.7	0.7 ± 0.0	1.0414	1.0457	80/99
25	489509098 *489972034*	Iron-dependent repressor IdeR *Dihydrofolate reductase*	0.1 ± 0.0	0.2 ± 0.0	–	1.0362	1.0409	93/100
26	489996564 *497664056*	D-3-phosphoglycerate dehydrogenase *Phosphoglycerate dehydrogenase*	0.1 ± 0.0	0.3 ± 0.0	–	1.0603	1.0551	89/100
27	489500222 *495529442*	Molybdenum cofactor biosynthesis protein *Molybdenum cofactor biosynthesis protein*	0.1 ± 0.0	0.1 ± 0.0	0.3 ± 0.4	1.0536	1.0476	96/91
28	554793963 *497660570*	Hypothetical protein O217_19000 *Hypothetical protein*	0.1 ± 0.0	0.4 ± 0.0	0.2 ± 0.2	1.0490	1.0422	85/89
29	489497388 *489970521*	Dihydrolipoyl dehydrogenase *Dihydrolipoyl dehydrogenase*	0.1 ± 0.0	0.1 ± 0.0	0.3 ± 0.3	1.0408	1.0343	90/99
30	489497373 *489970531*	hypothetical protein *hypothetical protein*	0.1 ± 0.0	1.2 ± 0.2	–	1.0212	1.0135	76/100
31	489997334 *497663425*	Phosphoglycerate kinase *Phosphoglycerate kinase*	0.1 ± 0.0	0.2 ± 0.0	–	1.0415	1.0428	82/88
32	489497373 *489970531*	Hypothetical protein *Hypothetical protein*	0.1 ± 0.0	1.2 ± 0.2	–	1.0209	1.0136	76/100

NCBI accession codes and protein names are reported in normal text for bPPD and in *italics* for aPPD

*AAP* average antigenic propensity

### Experimental comparison of the immunogenicity of bPPD, aPPD and P22

We hypothesised that P22 could offer greater specificity than bPPD in serodiagnosis because P22 shares far fewer proteins with aPPD, while still offering comparable sensitivity since P22 and bPPD share seven highly abundant proteins, five of which are predicted to be immunogenic (Tables 1, 3).

**Fig. 2:**
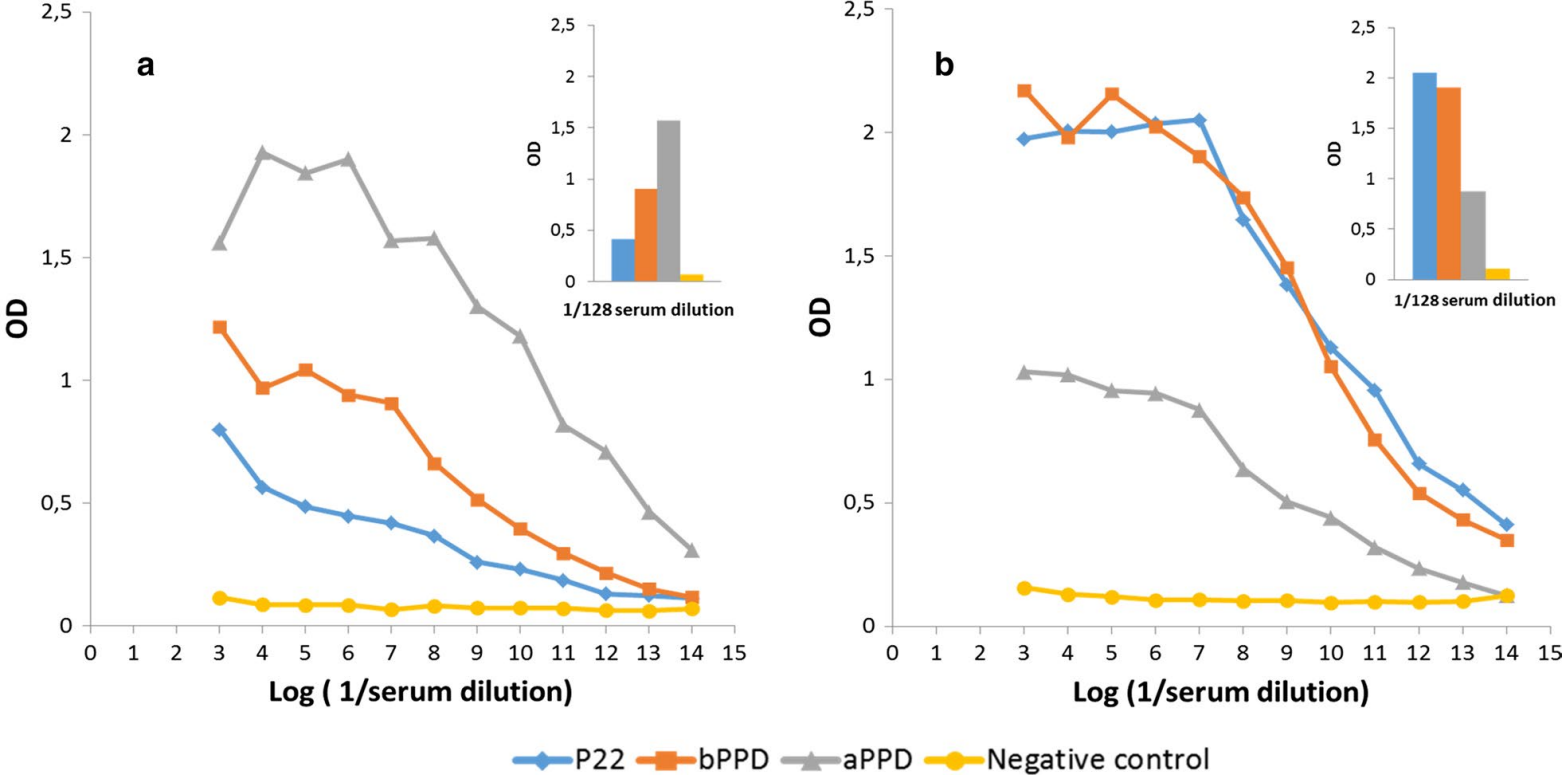
Indirect ELISA for serum reactivity from animals immunised with **a** aPPD or **b** bPPD against bPPD, aPPD and P22. As a negative control, serum was obtained from non-immunised mice (yellow line). The inset shows results obtained at serum dilutions of 1:128. *OD* optical density

We compared the ability of immunosera from mice hyperimmunised with bPPD or aPPD to recognise bPPD, aPPD and P22. Serum from mice immunised with aPPD reacted more strongly against aPPD and less strongly against bPPD and P22, indicating substantial cross-reaction (Fig. 2a). Similarly, serum from mice immunised with bPPD reacted against bPPD and P22, but it also cross-reacted with aPPD (Fig. 2b). These results suggest that, in the field, animals reacting against both aPPD and bPPD, particularly those reacting more strongly against aPPD, may be incorrectly categorised as negative. Using P22 in ELISA-type assays may avoid this problem, offering greater specificity and comparable or greater sensitivity than bPPD for tuberculosis serodiagnosis. Proof-of-concept surveys testing P22 for serodiagnosis of animal tuberculosis are ongoing. Casal et al. [20] showed that an antibody detection test significantly improved the sensitivity of in vivo bovine tuberculosis diagnosis. Hence, diagnostic techniques detecting both cellular and humoral responses may be an alternative for controlling bovine tuberculosis outbreaks in high-prevalence settings. Moreover, some antibody detection techniques have been shown to give the best performance in cattle with gross tuberculosis lesions [29].

We identified 32 highly abundant and immunogenic proteins that contribute to cross-reactivity between aPPD and bPPD (Table 4). These results are consistent with previous proteomics work highlighting the importance of the chaperones DnaK, GroEL, and GroES as well as elongation factor Tu and acyl carrier protein as sources of cross-reactivity [21, 22, 30]. Chaperones, which are conserved among most mycobacteria and other bacteria that cause respiratory disease [31], play important roles in humoral and cellular innate and adaptive immune responses [32, 33]. Our results, then, are consistent with work suggesting that false-positive serodiagnosis of tuberculosis tends to reflect immune responses due to homologous vaccination antigens or environmental mycobacteria [34, 35].

Our results here, based purely on antibody-based immune responses, suggest that P22 may be a superior alternative to bPPD. It would be important to verify this by experimentally assessing T cell-mediated immune responses, which cooperate with antibody-based responses to mount a collaborative defense [36]. We expect that T cell-mediated responses would be similar to the antibody-based responses described here because several of the most abundant proteins in bPPD are powerful T immunogens, including MPB70, MPB83, ESAT-6 and CFP-10 [37–42].

## Conclusion

On the basis of these results, we propose that a subpreparation of bPPD called P22 may be an alternative to bPPD for tuberculosis diagnosis, offering greater specificity as well as similar or even greater sensitivity for ELISA-type detection of antibodies against *M. tuberculosis* complex. P22 should be tested in field studies of tuberculosis serodiagnosis, and its ability to elicit T cell-mediated responses should be analysed, since it contains several antigens recognised by T cells, including MPB70, MPB83, ESAT-6 and CFP-10. In addition, we have analysed the protein composition of bPPD and aPPD, assessing the relative abundance and immunogenicity of major components. We identified several highly antigenic proteins specific to *M. bovis*, such as MPB70, MPB83, MPB63 and MPB64, which may therefore be the most useful in serological diagnosis of tuberculosis. We also identified several proteins common to bPPD and aPPD that may help explain the cross-reactivity between them in standard tuberculosis tests; these shared proteins include the chaperones GroES and DnaK, meromycolate extension acyl carrier protein, secretion protein and 50S ribosomal protein L7/L12.

## Additional files

**Additional file 1.** Peptides and proteins identified in bPPD.

**Additional file 2.** Peptides and proteins identified in aPPD.

**Additional file 3.** Peptides and proteins identified in P22.

**Additional file 4.** Proteins shared between bPPD and aPPD.

## Abbreviations

AAP: average antigenic propensity
aPPD: avian purified protein derivative
bPPD: bovine purified protein derivative
CFP-10: ESAT-6-like protein EsxB
IFN: interferon
SIT: single intradermal test
